# Chemical Lectures on Diseases of Nervous System

**Published:** 1874-11

**Authors:** 


### BIBLIOGRAPHICAL.

---

*Clinical Lectures on Diseases of the Nervous System.* By William A. Hammond, M. D., Professor of Diseases of the Mind and Nervous System in the University of the City of New York, etc., etc., etc. Reported and Edited, and the Histories of the Cases prepared and edited by T. M. B. Cross, M. D.

These Clinical Lectures were delivered at the New York State Hospital for Diseases of the Mind and Nervous System, and at Bellevue Hospital Medical College by Prof. Hammond, and are reported in full, together with the histories of the cases, by Dr Cross, after careful study and prolonged observation, with the hope that they may serve to add something to the clinical literature of nervous diseases.

The names of the author and compiler are sufficient to show the value of the work, which is gotten up in a handsome volume by the Publishers, D. Appleton & Co., New York.

